# Information filtering by synchronous spikes in a neural population

**DOI:** 10.1007/s10827-012-0421-9

**Published:** 2012-09-12

**Authors:** Nahal Sharafi, Jan Benda, Benjamin Lindner

**Affiliations:** 1 Max-Planck-Institut für Physik komplexer Systeme, Dresden, Germany; 2 Biozentrum München, Bernstein Center, Munich, Germany; 3 Institute for Neurobiology, University Tübingen, Tübingen, Germany; 4 Present Address: Bernstein Center for Computational Neuroscience Berlin, Berlin, Germany; 5 Institute of Physics, Humboldt University Berlin, Berlin, Germany; 6 Present Address: Max Planck Institute for Dynamics and Self-Organization, Bunsenstrasse 10, 37073 Göttingen, Germany

**Keywords:** Synchrony, Neural coding, Stochastic integrate-and-fire neuron, Noise

## Abstract

Information about time-dependent sensory stimuli is encoded by the spike trains of neurons. Here we consider a population of uncoupled but noisy neurons (each subject to some intrinsic noise) that are driven by a common broadband signal. We ask specifically how much information is encoded in the synchronous activity of the population and how this information transfer is distributed with respect to frequency bands. In order to obtain some insight into the mechanism of information filtering effects found previously in the literature, we develop a mathematical framework to calculate the coherence of the synchronous output with the common stimulus for populations of simple neuron models. Within this frame, the synchronous activity is treated as the product of filtered versions of the spike trains of a subset of neurons. We compare our results for the simple cases of (1) a Poisson neuron with a rate modulation and (2) an LIF neuron with intrinsic white current noise and a current stimulus. For the Poisson neuron, formulas are particularly simple but show only a low-pass behavior of the coherence of synchronous activity. For the LIF model, in contrast, the coherence function of the synchronous activity shows a clear peak at high frequencies, comparable to recent experimental findings. We uncover the mechanism for this shift in the maximum of the coherence and discuss some biological implications of our findings.

## Introduction

In many sensory modalities, time-varying stimuli are encoded by a population of many neurons (Gollisch and Meister 2008; Clemens et al. 2011; Vonderschen and Chacron 2011). Examples of such populations are found in common visual (Knight 1972b; Wandell 1995) and auditory systems (Hudspeth 2000) of many organisms, but also in the more exotic electrosensory system of electric fish (Heiligenberg 1991; Krahe et al. 2008). The simplest case with relevance e.g. for auditory and electrosensory signal processing is certainly that of a neural population without lateral connections among the cells but with an overlap in their receptive fields. Theoreticians have explored this simple setup mainly in population models of uncoupled neurons which are subject to intrinsic fluctuations (e.g. channel noise or synaptic background noise) and to common input stimulus (arising from the overlap in the receptive fields) (Knight 1972a; Stocks and Mannella 2001; Gerstner and Kistler 2002).

Even if one focusses only on the mean activity of the whole population (the population rate), the simple case of uncoupled neurons with common noise holds surprises. Intrinsic fluctuations of the neurons, for instance, can play a constructive role for the signal transmission. Individual noise for each neuron is needed in such populations to escape entrainment by certain stimulus frequencies and thus to achieve a reasonable representation of the stimulus in the population firing rate (Knight 1972a). For a given stimulus amplitude a specific non-zero level of the intrinsic noise optimizes the mutual information between the common stimulus and the summed spiking activity of the population (Stocks and Mannella 2001).

However, there are additional ways in which information can be encoded in the activity patterns of populations of neurons, for instance, synfire chains (Abeles 1991) resulting in precise spike pattern, so-called unitary events (Grün 2009), network oscillations that permit phase precession in place cells (OKeefe and Recce 1993), or the synchronisation-desynchronization code proposed by (Benda et al. 2006). Synchronous activity can be easily read out by coincidence detectors (Softky and Koch 1993). In the above mentioned case of uncoupled neurons, the cells have no lateral interaction and, consequently, synchrony in their activity is solely stimulus-driven. Put differently, these neurons fire together if they belong to the same receptive field and the sensory stimulus is strong enough to overcome the desynchronizing effect of intrinsic noise, which is independently at work in each of the neurons.

Recently, Middleton et al. (2009) investigated the information carried by synchronous spikes about a stimulus in P-unit afferents of weakly-electric fish. They found that the synchronous spikes preferentially encode information about high-frequency stimuli in contrast to the information of all spikes that is more broad-band. Information transmission was measured in terms of the spectral coherence function that directly shows in which frequency band the information flux is maximized. Although the experimentally observed maximum of the coherence function at high stimulus frequencies was also confirmed in simulations of models of rather different levels of complexity, the mechanism for this shaping of information transmission is still poorly understood.

In this paper, we study analytically the problem of signal transfer by synchronous activity in a neural population. We develop a mathematical framework for characterizing synchrony by the product of Gaussian convolutions of spike trains in Section 2.1. We then calculate the linear response for this ’product spike train’ and derive in Section 2.2 formulas for the mean value and the power spectrum of the synchronous spikes as well as their cross-spectrum with the time-dependent stimulus. We apply our general results to two neuron models: the inhomogeneous Poisson process (Section 3.1) and the leaky integrate-and-fire model with current noise and a time-dependent stimulus (Section 3.2) and show under which circumstances the coherence of the synchronous spikes can be indeed high-pass filtered. We discuss how the coherence depends on the number of spike trains involved in the product and how its peak frequency changes under strong stimulation (nonlinear response, determined here not analytically but purely by simulations). Finally, we summarize our results and discuss implications for neural coding in Section 4.

## Materials and methods

### Model, measures of synchrony, and measures of signal transmission

**Fig. 1:**
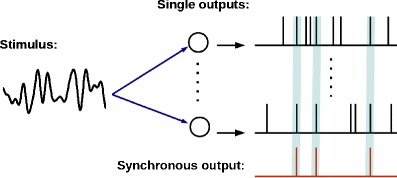
Model of *n* noisy neurons, which are driven by a common broadband noise. Given the spikes generated by the single neurons (*upper* time traces), the *synchronous output* of the population (*lower* time trace) shows spikes only if all of the neurons fire a spike in close temporal neighborhood. The main question in this paper is, how much information the synchronous output carries about the common stimulus in different frequency bands

We consider a population of *n* uncoupled spiking neurons, each of which is subject to some independent fluctuations (intrinsic noise, e.g. ion channel noise) leading to stochastic spiking. In addition, the population is stimulated by a common broadband Gaussian signal (also called stimulus in what follows); cf. Fig. 1. Specifically, we use a Gaussian-white-noise stimulus with cut-off frequency *f*_*c*_ and a power spectrum

1 $$ S_{s,s}(f) = \left\{ {\begin{array}{l l} 2 D_s & ,-f_c \leq f \leq f_c \\ 0 & ,\text{otherwise.} \\ \end{array}} \right. $$

The intensity of the stimulus signal, *D*_*s*_, is given by half of the height of the spectrum at *f* = 0. Because the general theory does not hinge on the exact shape of the input spectrum, we will use the more general expression *S*_*s*,*s*_(*f*) in our derivations and formulas and only for numerical evaluations and simulations employ Eq. (1).

We are interested in the synchronous output, i.e. in the unitary events when all *n* neurons fire (within a certain temporal resolution) “at the same time”. In the experiment by Middleton et al. (2009), the synchronous output was defined by means of an algorithm that works as follows (cf. Fig. 1) : one of the spike trains is singled out as the reference spike train (RST). Each spike in the RST is surrounded by a time window of size *τ*. Only if within this time window we find a spike in all the *n* − 1 other spike trains of the population, only in this case, a spike in the synchronous output is generated. This spike will be assigned to the time instant of the reference spike in the RST. From the theoretical point of view, measuring the synchronous output in this way is somewhat inconvenient and we will now introduce a more tractable but still related function.

We choose the following definition of the synchronous output. In a first step, all *n* spike trains *x*_*k*_(*t*) = ∑ *δ*(*t* − *t*_*k*,*i*_) are convolved with a smoothing kernel:

2 $$ y_k(t)=F* x(t)=\int_{-\infty}^\infty dt' x_k(t') F(t-t') . $$

The kernel *F*(*τ*) is a Gaussian throughout this work:

3 $$ F(\tau)=\frac{1}{\sqrt{2\pi \sigma^2}} e^{-\frac{\tau^2}{2\sigma^2}} . $$

As for the power spectrum of the stimulus, our general theory does not depend on the Gaussian property of the filter (although the simplifications done for a population of Poisson neurons do); in the calculations of the next section we will thus keep the more general expression for the filter and its Fourier transform (for the latter, see below).

The convolution will still resemble the original spike train if the width of the Gaussian is small compared to the typical interspike interval (ISI), i.e. to the inverse firing rate *r*:

4 $$ r \sigma \ll 1. $$

Below, if not stated otherwise, we will use values of *σr* = 0.1 for the Poisson case and *σr*_0_ = 0.07 for the LIF neurons.

As a measure of synchrony, we use the product of all these convolved spike trains, normalized in such a way that for perfectly aligned spikes at a certain time *t*, the resulting Gaussian peak is normalized to one:

5 $$ y_{\rm SO}(t)=\alpha \prod_{k=1}^n y_k(t), \;\; \alpha=\sqrt{n}(2\pi\sigma^2)^{\frac{n-1}{2}} . $$

**Fig. 2:**
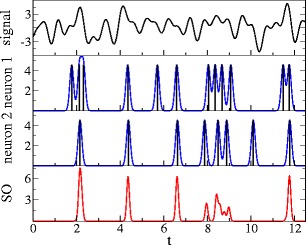
Synchronous output by multiplication of Gaussian convolutions of spike trains in a population of leaky integrate-and-fire neurons (here: two neurons). Parameters: *μ* = 0.9, *D* = 0.8 and *D*_*s*_ = 0.24 (for details on the model and definitions of parameters, see Section 3.2)

Obviously, *y*_SO_(*t*) is not a spike train anymore but a continuous function of time. However, for sufficiently small values of *σ*, the product of Gaussians will only be finite at time *t* if all the *n* neurons have spiked about *t* (Fig. 2).

Because we will consider neurons with intrinsic noise that are driven by a common (also noisy) stimulus *s*(*t*) (also called *signal* in what follows), we have to deal with two different statistical ensembles and, consequently, two distinct averages. We will denote the average with respect to the stimulus by an index (${\langle\cdot\rangle}_s$) while averages without index ($\langle{\cdot}\rangle$) are taken over the intrinsic noise. For the sake of illustration, consider, for instance, averages of the spike train. The stimulus-induced time-dependent modulation of the firing rate, is obtained by an average over only the intrinsic noise

6 $$ \langle{x(t)}\rangle =r(t) $$

which is, according to linear-response theory (Risken 1984; Rieke et al. 1996; Fourcaud and Brunel 2002) for a weak signal given by

7 $$ r(t)=r_0+K*s(t). $$

Here *r*_0_ is the firing rate of the isolated single neuron (only subject to its intrinsic noise) and the convolution with the linear-response function *K*(*t*) describes the linear modulation of the firing rate by a time-dependent stimulus. The linear-response approximation implies neglecting any rectification and saturation effects of the neural dynamics, which is justified for sufficiently small signal strength.

Averaging the spike train additionally with respect to the mean-zero signal yields in this approximation

8 $${\langle{\langle{x(t)}\rangle}\rangle}_s ={\langle{r(t)}\rangle}_s=r_0, $$

i.e. the average firing rate of the isolated cell.

In the following we will work mainly in the frequency domain and denote by a tilde the Fourier transformation. For numerical simulations, we use a finite-time-window version of the transform defined by

9 $$ \tilde{x}_T(f)=\int_{-T/2}^{T/2} dt\; x(t) e^{i2\pi f t}, $$

where *T* is the size of the time window which is centered around *t* = 0. Given, for instance, the spike train *x*(*t*) and the stimulus *s*(*t*), the power spectrum of *x*(*t*) and the cross-spectrum of spike train and signal are defined by

10 $$ \begin{array}{rll} S_{x,x}(f)&=& \lim\limits_{T\to\infty} \frac{{\langle{\langle{\tilde{x}_T(f)\tilde{x}_T^*(f)}\rangle}\rangle}_s}{T},\\ S_{x,s}(f)&=& \lim\limits_{T\to\infty} \frac{{\langle{\langle{\tilde{x}_T(f)\tilde{s}_T^*(f)}\rangle}\rangle}_s}{T} , \end{array} $$

where the asterisk in the superscript denotes the complex conjugated. In practice, the large-time limit implies that we use sufficiently large time windows that ensure a satisfying frequency resolution.

In the theoretical calculations it turns out to be advantageous to use the infinite-time transform

11 $$ \tilde{x}(f)=\int_{-\infty}^{\infty} dt\; x(t) e^{i2\pi f t}; $$

by means of which power and cross-spectra are indirectly defined via (Stratonovich 1967)

12 $$ \begin{array}{rll} {\langle{\langle{\tilde{x}(f)\tilde{x}^*(f')}\rangle}\rangle}_s&=&\delta(f-f') S_{x,x}(f),\\ {\langle{\langle{\tilde{x}(f)\tilde{s}^*(f')}\rangle}\rangle}_s&=&\delta(f-f') S_{x,s}(f). \end{array} $$

Note that we do not remove the DC part from spectral measures because they play an important and nontrivial role in our derivations.

In our calculations, we will also need the Fourier transform of the Gaussian filter *F*(*t*), which reads explicitely

13 $$ \tilde{F}(f)=\exp[-\beta f^2], $$

where *β* = 2*π* ^2^ *σ* ^2^.

For Gaussian signals, as used in this study, the information transmission of a system with output *x*(*t*) driven by a signal *s*(*t*) can be quantified in terms of the spectral input–output statistics. Specifically, we will employ the spectral coherence function, defined for positive frequencies (*f* > 0)

14 $$ C_{x,s}(f)=\frac{|S_{x,s}(f)|^2}{S_{x,x}(f)S_{s,s}(f)} , $$

where *S*_*x*,*s*_ is the cross-spectrum between signal and output and *S*_*x*,*x*_ and *S*_*s*,*s*_ are the power spectra of input and output, respectively. Unlike the cross-spectrum, the coherence is a nondimensional correlation coefficient (taking the range 0 < *C*(*f*) < 1) in the frequency domain that quantifies how much of the observed variability is shared between input and output. The coherence is related to a lower bound on the mutual information rate, which is the central quantity in the theory of information by Shannon (1948). The lower bound is given by an integral over the bandwidth *f*_*c*_ of the signal (Gabbiani 1996)

15 $$ I_{\rm LB}=-\int_0^{f_c} df \log_2[1-C(f)]. $$

From this formula we see that the contribution of the coherence at a certain frequency to the total information is a monotonic function of *C*(*f*)—the closer this function is to one, the more information is transmitted.

The frequency dependence of the coherence function gives us some idea whether the system under study preferentially encodes information about slow components of the signal (coherence is large at low but small at high frequencies), about fast components (coherence large at high but small at low frequencies), or whether information on all components are equally transmitted. Put differently, the shape of the coherence function tells us about information filtering in a system; see Lindner et al. (2009) for an application of this idea in the context of short-term synaptic plasticity. Note that the concept of information filtering has nothing to do with the usual power filtering seen in the transfer function or susceptibility of a system. A linear system with white input signal and white background noise does not filter the information (the coherence is flat), irrespective of the shape of the transfer function (low-pass, band-pass or high-pass filter with respect to spectral power).

In the following, we are specifically interested in how much information the synchronous output *y*_SO_(*t*) carries about the signal with respect to different frequency bands. To this end, we use the spectral coherence function of signal and synchronous output

16 $$ C_{{\rm SO},s}(f)=\frac{|S_{{\rm SO},s}(f)|^2}{S_{{\rm SO},{\rm SO}}(f)S_{s,s}(f)}. $$

In particular, we want to compare this to the coherence for a single (convolved) spike train

17 $$ C_{y,s}(f)=\frac{|S_{y,s}(f)|^2}{S_{y,y}(f)S_{s,s}(f)} = C_{x,s}(f). $$

Note that (as we indicated by the last equation above) the coherence of original and convolved spike trains agree. For better comparison of cross- and power spectra, we will however, solely consider spectral measures for the convolved spike train.

It is also interesting to compare the two coherence functions *C*_SO,*s*_ and *C*_*y*,*s*_ to a third one that includes all the available information of the output, namely, the sum of all the spike trains (“summed spike train”)

18 $$ Y(t)=\sum\limits_{k=1}^n y_k(t), $$

the coherence of which

19 $$ C_{Y,s}(f)=\frac{|S_{Y,s}(f)|^2}{S_{Y,Y}(f)S_{s,s}(f)} $$

is an upper bound to both single-spike-train coherence and coherence of the synchronous output. Frequency-dependent differences among the coherence functions can be understood based on the behavior of cross- and power spectra.

Before we start with the inspection of these second-order statistics, it is important to understand how in our setup the mean value of the synchronous output depends on the systems parameters (number of neurons, stimulus strength, etc.). This is important because the coherence of a neuron depends strongly on its firing rate, and the mean value of the synchronous output can be regarded (in loose analogy to the mean value of a spike train) as the synchronous firing rate. For these reasons, we will first approach the problem of calculating the mean value $\langle{y_{\rm SO}}\rangle$.

In the next section, we work out a linear-response theory for our general setup which holds true for weak common stimulus and sufficiently strong intrinsic noise within the individual neurons. We then apply our results to two neuron models, the Poisson model and the leaky integrate-and-fire neuron model.

### General theory for spectral measures of the synchronous output

A central ansatz by Lindner et al. (2005b) used previously for a theory of spectral measures in neural networks with delay assumes that the single spike train’s Fourier transformation obeys a linear response with respect to an external signal:

20 $$ \tilde{x}(f) = \tilde{x}_0(f) + \chi (f) \tilde{s} (f). $$

Here, $\chi(f)=\tilde{K}(f)$ is the susceptibility, i.e. the Fourier transform of the linear response function *K*(*t*). In the time domain, our ansatz reads

21 $$ x(t) = x_0(t) + K * s (t). $$

In particular in the time domain, this linear ansatz seems to be somewhat doubtful because *x*_0_(*t*) is a spike train and the signal will change the timing of the spikes and will not add a continuous contribution as suggested by Eq. (21). We emphasize, however, that the equation is correct on average (cf. Eq. (7)). Furthermore, the ansatz Eq. (20) has been successfully employed for calculating spectral measures in neural networks (Lindner et al. 2005b; Marinazzo et al. 2007; de la Rocha et al. 2007; Shea-Brown et al. 2008). We will show in this paper that it also works well for characterizing the signal transfer by the SO, provided the common stimulus *s*(*t*) is sufficiently weak.

Before we approach this problem, we first discuss how the mean value of the SO depends on population size and how strong the firing rate is modulated by the common stimulus.

### Mean value (firing rate) of the synchronous output

As pointed out before, for small *σr* it makes sense to interpret the mean value of the synchronous output as the synchronous firing rate *r*_SO_, i.e. this mean value gives us an idea about the number of synchronous events per unit time. The mean value over the two ensembles yields

22 $$ \begin{array}{rll} r_{\rm SO}=\langle\langle y_{\rm SO}(t) \rangle\rangle &=& \langle\langle \alpha \prod\limits_{k} y_{k}(t) \rangle\rangle_{s} \\ &=& \alpha \langle (F*r(t))^n \rangle_s. \end{array} $$

Using Eq. (7), we arrive at the following expression for the firing rate of the SO:

23 $$ r_{\rm SO} = \alpha \langle (r_0 + F*K*s(t))^n \rangle_s . $$

Because the convolution is a linear operation and the stimulus is Gaussian, the function $\hat{s}(t)= F*K*s(t)$ appearing in Eq. (23) will be Gaussian as well with a variance $\langle{\hat{s}^2}\rangle$, given by the integral over its power spectrum. The latter is simply the product of the signal’s power spectrum and the absolute squares of filter and susceptibility:

24 $$ {\langle{\hat{s}^2}\rangle}_s=\int_{-\infty}^{\infty}|\tilde{F}(f)\chi(f)|^2 S_{s,s}(f) \, df. $$

Once this variance has been calculated, we can perform the average in Eq. (23) by integrating over a Gaussian

$$ \begin{array}{rll} r_{\rm SO} &=& \alpha r_0^n \int_{-\infty}^{\infty} du \, \frac{e^{-\frac{u^2}{2{\langle{\hat{s}^2}\rangle}_s}}}{\sqrt{2\pi{\langle{\hat{s}^2}\rangle}_s}} (1+u/r_0)^n \\ &=& \alpha r_0^n \sum\limits_{k=0}^n \begin{pmatrix} n \\ k \end{pmatrix} \int_{-\infty}^{\infty} du \, \frac{e^{-\frac{u^2}{2{\langle{\hat{s}^2}\rangle}_s}}}{\sqrt{2\pi{\langle{\hat{s}^2}\rangle}_s}} \left(\frac{u}{r_0}\right)^k . \end{array} $$

Using the fact that averages over odd powers vanish and changing the summation index such that it extends to $\lfloor n/2\rfloor$ (the largest integer not greater than *n*/2), we arrive at the final formula

25 $$ \begin{array}{rll} r_{\rm SO} & = & r_0 \sqrt{2 n (2\pi)^{n-2}} (r_0\sigma)^{n-1} \\ && \times \sum\limits_{k=0}^{\lfloor n/2\rfloor} \begin{pmatrix} n \\ 2 k \end{pmatrix} \Gamma \left(\frac{1}{2} + k \right) \left(\frac{2{\langle{\hat{s}^2}\rangle}_s}{r_0^{2}}\right)^k . \end{array} $$

This formula permits the following conclusions. The dependence on the relative width *r*_0_ *σ* of the Gaussian filter (i.e. the ratio of width *σ* and mean interspike interval $r_0^{-1}$) is exponential in the population size; $r_{\rm SO} \sim (r_0 \sigma)^{n-1}$, no matter what neuron model is considered and whether a stimulus is present or not. Furthermore, properties of the neuron may affect the rate only via the effective variance Eq. (24) that is shaped by the neuron’s susceptibility *χ*. For vanishing signal, the synchronous rate reads

26 $$r_{{\rm SO},0}= r_0 \sqrt{n (2\pi)^{n-1}} (r_0\sigma)^{n-1} . $$

It is interesting to note that for sufficiently large relative width the synchronous rate may *increase* with *n*; *r*_SO,0_ for two neurons (*n* = 2), for instance, is smaller than the isolated rate *r*_0_ (i.e. *r*_SO,0_ for *n* = 1) only if

27 $$ r_0\sigma<\frac{1}{2\sqrt{\pi}}; $$

otherwise, the synchronous rate increases for small *n*. However, with our choice *r*_0_ *σ* = 1/10, the above inequality is always fulfilled and thus the rate drops with increasing *n*.

For weak stimulation, it suffices in Eq. (25) to take into account only the term proportional to the variance ${\langle{\hat{s}^2}\rangle}_s$. This yields a stimulus-induced increase in the rate by a factor of

28 $$ \frac{r_{\rm SO}}{r_{{\rm SO},0}} \approx \left(1+ \frac{n(n-1)}{2} \frac{{\langle{\hat{s}^2}\rangle}_s}{r_0^2} \right) , $$

which becomes more important for larger populations (note, however, the exponential decrease of the SO firing rate with increasing *n*, given by the prefactor).

### Cross-spectra of outputs with the common stimulus

For the single spike train, we find as expected:

29 $$ \begin{array}{rll} {\langle{\langle \tilde{y}(f) \tilde{s}^{\ast}(f') \rangle}\rangle}_s &=& \tilde{F}(f) {\langle{\langle [\tilde{x}_0(f) + \chi (f) \tilde{s} (f)]\tilde{s}^{\ast}(f')\rangle}\rangle}_s\\ &=&\tilde{F}(f) \chi (f) {\langle{s(f) s^*(f')}\rangle}_s . \end{array} $$

By means of Eq. (12), we thus find the cross-spectrum of stimulus and single spike train to be given by

30 $$ S_{y,s}(f)= \tilde{F}(f) \chi (f)S_{s,s}(f) . $$

For the sum of the spike trains, the cross-spectrum is just the sum of the cross-spectra between single spike train and stimulus, hence for *n* identical neurons, we have

31 $$ S_{Y,s}(f)= n \tilde{F}(f) \chi (f)S_{s,s}(f)=nS_{y,s}(f). $$

In order to calculate the cross-spectrum of the synchronous output, it is helpful to express the output’s Fourier transform as an *n*-fold convolution of the *n* spike trains

$$ \tilde{y}_{\rm SO}(f) = \alpha \underbrace{\tilde{y}_1(f)\ast \tilde{y}_2(f)\ast \cdots \ast \tilde{y}_n(f)}_{\text{n-times}} . $$

Writing the convolution integrals explicitly, we obtain

32 $$ \begin{array}{rll} &&{\langle{\langle \tilde{y}_{\rm SO}(f) \tilde{s}^{\ast}(f^{\prime})\rangle}\rangle}_s = \alpha \int \ldots \int d f_1 \ldots df_{n-1} \\ && \tilde{F}(f_{1})\ldots \tilde{F}(f_{n-1}) \tilde{F}(f-f_{1}\ldots -f_{n-1}) \times \langle \tilde{s}^{\ast}(f^\prime) \\ &&[r_{0}\delta(f_{1})+\chi(f_{1}) \tilde{s}(f_{1})] \cdots [r_{0}\delta(f_{n-1})+\chi(f_{n-1})\tilde{s}(f_{n-1})] \\ &&[r_{0}\delta(f-\ldots f_{n-1}) +\chi(f-\ldots f_{n-1})\tilde{s}(f-\ldots f_{n-1})]\rangle_s . \end{array} $$

For a weak stimulus, the two strongest contributions to the cross-spectrum read

33 $$ \begin{array}{rll} S_{{\rm SO},s}(f) &\simeq& \alpha n r_{0}^{n-1} \tilde{F} \chi S_{s,s} +\alpha r_0^{n-3} \frac{n(n-1)(n-2)}{2}\\ && \times\tilde{F}\chi S_{s,s} \int \tilde{F}^2(f_1)|\chi(f_1)|^2 S_{s,s}(f_1) \, df_1. \end{array} $$

For the ease of notation, here and in the following we omit the frequency argument *f* from filter, susceptibility, and spectrum if it is not an integration variable. Higher-order contributions to the cross-spectrum will involve multidimensional integrals over the stimulus spectrum, response function, and the neuron’s susceptibility. An important conclusion from Eq. (33) is that to lowest order the cross-spectrum of the synchronous output shares the frequency dependence of that of the single spike train given by $\tilde{F} \chi S_{s,s}$.

### Power spectra

With our assumption of a realization-wise linearity of the input–output relation, it is easy to calculate the power spectrum of the single filtered spike train (for a critical evaluation of this approximation of the spectrum, see Lindner et al. (2005a)). In terms of the unperturbed power spectrum $S_{x_0,x_0}$ (in the absence of a stimulus), response function *χ*, and driving spectrum *S*_*s*,*s*_, the power spectrum of *y*(*t*) reads

34 $$ S_{y,y} = |\tilde{F}|^2\big[S_{x_0,x_0} +|\chi|^2 S_{s,s}\big], $$

where $S_{x_0,x_0}$ is the power spectrum of the spontaneous spike train and, correspondingly,

35 $$ S_{y_0,y_0} = |\tilde{F}|^2 S_{x_0,x_0} $$

is the power spectrum of the filtered spontaneous spike train. From Eq. (34) it is evident that for a weak signal the leading contribution to the spectrum comes from the unperturbed power spectrum.

For the calculation of the spectrum of the summed spike train, one has to take into account that the unperturbed spike trains are statistically independent (yielding only *n* non-vanishing terms) but the stimulus dependent parts are not (yielding *n* ^2^ finite terms):

36 $$ S_{Y,Y} = |\tilde{F}|^2 [n S_{x_0,x_0} + n^2 |\chi(f)|^2 S_{s,s}] . $$

Compared to the spectrum of the single spike train, the stimulus makes a far stronger contribution, in particular, for large networks with *n* ≫ 1. In the case of interest here, in which we only consider a few neurons, we may still assume that for a weak stimulus the spontaneous spectrum dominates the power spectrum.

The synchronous output’s power spectrum reads

37 $$ \begin{array}{rll} && \langle \langle \tilde{y}_{\rm SO}(f)\tilde{y}^*_{\rm SO}(f') \rangle \rangle_s =\alpha ^2 \left\langle \int df_1 df_1^\prime \ldots df_{n-1}df^{\prime}_{n-1}\right. \\ && \langle \tilde{y}_1(f_1) \tilde{y}^{\ast}_1(f^{\prime}_1)\rangle \ldots\langle \tilde{y}_{n-1}(f_{n-1})\tilde{y}^{\ast}_{n-1}(f^{\prime}_{n-1})\rangle \\ && \left.\frac{}{} \langle \tilde{y}_n(f-f_1 \ldots f_{n-1}) \tilde{y}^{\ast}_n (f'-f^{\prime}_1 \ldots -f^{\prime}_{n-1}) \rangle \right\rangle_{s} . \end{array} $$

Here products involving the same spike train have been treated as statistically independent of other products within the ${\langle{\cdots}\rangle}_s$ average; in this picture, *s*(*t*) corresponds to a frozen stimulus. The product (not averaged over the stimulus) of the spike train’s Fourier transforms can be expressed as follows:

38 $$ \begin{array}{rll} &&\langle \tilde{y}(f)\tilde{y}^*(f')\rangle = \tilde{F}(f)\tilde{F}^*(f')\big\{\delta(f-f')S_{x_0,x_0}(f)+\\ && r_0\left[\frac{}{}\delta(f')\chi(f)\tilde{s}(f)+\delta(f)\chi^*(f')\tilde{s}^*(f')\right] \\ && \qquad\qquad\qquad\qquad\qquad +\chi(f)\chi^*(f')\tilde{s}(f)\tilde{s}^*(f')\big\} . \end{array} $$

To zeroth order ($\langle{|\tilde{s}|^2}\rangle=0$), this yields, if inserted above, that the power spectrum of the synchronous output is proportional to an *n*-fold convolution of the spontaneous power spectrum $S_{y_0,y_0}$ (the latter was given in Eq. (35)). As can be shown, the next order correction in $\langle{|\tilde{s}|^2}\rangle$ is proportional to the stimulus power spectrum and an *n* − 1-fold convolution of $S_{y_0,y_0}$. For a weak stimulus, the power spectrum of the synchronous output reads

39 $$ \begin{array}{rll} &&{\kern-6pt} S_{{\rm SO},{\rm SO}} \simeq \alpha^2 \left[\frac{}{}\underbrace{S_{y_0,y_0} * \cdots * S_{y_0,y_0}(f)}_{\text{n-times}} \right. \\ &&{\kern-6pt}\left. +\, n (|\tilde{F}\chi|^2 S_{s,s}) \ast \underbrace{S_{y_0,y_0} \ast \ldots \ast S_{y_0,y_0}(f)}_{\text{(n-1)-times}} \right. \\ &&{\kern-6pt} \left. + \frac{}{} 2 n(n-1) r_0^2 (|\tilde{F}\chi|^2 S_{s,s}) \ast \underbrace{S_{y_0,y_0} \ast \ldots \ast S_{y_0,y_0}(f)}_{\text{(n-2)-times}})\right]. \end{array} $$

Note that the unperturbed power spectrum still includes a DC peak (a delta peak at zero frequency).

### Coherence function

As pointed out before, the spectral coherence is essentially formed by the ratio of squared transferred signal power and power spectrum. From the theoretical results achieved so far, we can draw already some conclusions about differences of the signal coherence for a single neuron and for the synchronous output.

If we restrict ourself to very weak stimulation, we find for the cross-spectrum from Eqs. (30) and (33)

40 $$ S_{{\rm SO},s} \simeq \alpha n r_{0}^{n-1} \tilde{F}(f) \chi S_{s,s}(f) \simeq \alpha n r_{0}^{n-1} S_{y,s}(f) . $$

Because the prefactor on the right hand side does not depend on frequency, this proves that the cross-spectrum of signal and SO is to lowest order in signal power just a rescaled version of what we would observe for the single spike train. Any change in the frequency dependence of the coherence between single neuron and synchronous output (or between ‘all spikes’ and synchronous output) cannot be related to what happens in the numerator (squared cross-spectrum) but must be thus due to the frequency dependence of the denominator, that is, the power spectrum.

Indeed, for the power spectrum we find to lowest order (no stimulus) a qualitative difference: while for single and summed spike train, the power spectrum Eqs. (34) and (36), respectively, is proportional to the power spectrum of the unperturbed system $S_{y_0,y_0}=|\tilde{F}|^2S_{x_0,x_0}$, for the power spectrum of the synchronous output, this is rather proportional to the *n*-fold convolution of $S_{y_0,y_0}$ Eq. (39). We will now explore what the consequences of this convolution is in the cases of Poisson neurons and of LIF neurons.

## Results

### Population of Poisson neurons

The first model of the single neuron is the inhomogeneous Poisson process. The spike train of the *k*th neuron occurs with a time-dependent rate

41 $$ r_k(t)=r_0[1+ s(t)] . $$

Apart from the common rate modulation, spikes of different neurons are independent of each other. The signal *s*(*t*) is a Gaussian bandpass-limited white noise with variance $\langle{s^2}\rangle$ that we take to be much smaller than 1, in order to avoid a trivial rectification effect: for strong modulation, the rate *r*(*t*) will go to negative values, which cannot affect the spike generator and cannot be properly encoded in the sequence of spikes. To simplify some of the formulas, we will also assume a cut-off frequency *f*_*c*_ that is much larger than that of the Gaussian filter *σ* ^− 1^. Information transmission through a Poisson model has been studied analytically by different authors (Bialek and Zee 1990; Gabbiani 1996; Goychuk 2001).

Because in our model the rate is directly proportional to the signal *s*(*t*), the susceptibility is just a real constant:

42 $$ \chi(f)=r_0 . $$

**Fig. 3:**
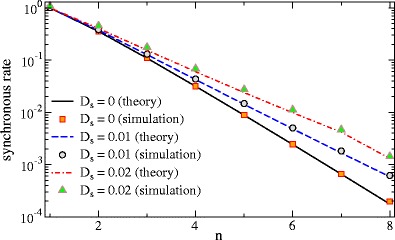
Mean of the synchronous output (“synchronous firing rate”) vs number of spike trains for different amplitudes of the signal as indicated and a cut-off frequency of *f*_*c*_ = 10

For a Gaussian filter (Eq. 3), a band-pass limited white noise with cut-off frequency *f*_*c*_, and the constant susceptibility, we can calculate the effective variance (Eq. 24):

43 $$ \begin{array}{rll} {\langle{\hat{s}^2}\rangle}_s&=&r_0^2 S_{s,s} \int_{-f_c}^{f_c} df \exp[-4\pi^2\sigma^2 f^2]\\ &=&\frac{r_0^2 D_s}{\sigma\sqrt{\pi}} \mbox{erf}(2\pi\sigma f_c) \approx \frac{r_0^2 D_s}{\sigma\sqrt{\pi}}. \end{array} $$

where in the last step we used the assumption of a high cut-off frequency. Inserting this into our general expression for the rate (Eq. 25), we can compare the resulting formula to simulation results (cf. Fig. 3).

As expected the rate drops with increasing number of neurons (spike trains) and a common stimulus *s*(*t*) amplifies the rate, in particular at higher number of neurons. The agreement of the simulations and the theory is good.

Next, we compare the spectral measures for single spike train, summed spike train, and SO. For the single neuron, we obtain for *f* < *f*_*c*_ and using Eq. (13):

44 $$ S_{y,s}(f) = 2 D_s r_{0}e^{-\beta f^2} $$

45 $$ S_{y,y}(f) = (r_{0} + 2 r_{0}^2 D_s+r_0^2 \delta(f)) e^{-2\beta f^2} $$

46 $$ C_{y,s}(f) = \frac{2 r_{0} D_s }{(1+ 2 r_{0} D_s)}; $$

we recall that *β* = 2*π* ^2^ *σ* ^2^.

In order to obtain the respective statistics for the summed output, we would have to replace *r*_0_ simply by *nr*_0_.

**Fig. 4:**
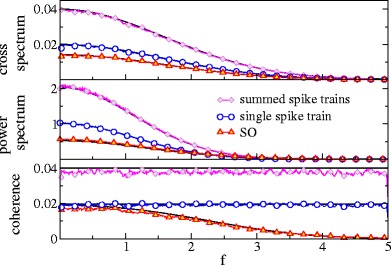
Spectral measures of information transmission in a population of Poisson neurons. Cross-spectra, power spectra and coherence of the stimulus with the single spike train (*blue*), with the summed output (*magenta*), and with the synchronous output (*red*). Simulation results are compared to the theoretical expressions (*black*) obtained from Eqs. (44–47, 31, 36, 19) for single and summed spike trains and from Eqs. (47, 51, 16) for the synchronous output. Parameters: *n* = 2 , *r*_0_ = 1, *D*_*s*_ = 0.01, *f*_*c*_ = 5

Although due to the effect of filtering by *F* the cross- as well as the power spectra of the single spike train and the summed output show a low-pass behavior, the coherence function is spectrally flat because the frequency dependencies of both spectra cancel (cf. Fig. 4). Of course, the coherence of the summed output is generally larger: for a weak signal, the coherence of *Y* is just the *n*-fold coherence of the single spike train.

For the SO, the cross-spectrum (33) reads

47 $$ S_{{\rm SO},s}(f) = \frac{2 n^{3/2}r_0^n D_s}{(2\pi\sigma^2)^{\frac{1-n}{2}}} e^{-\beta f^2} \left[1 + \frac{(n-1)(n-2)D_s}{\sqrt{2\beta/\pi}}\right] , $$

where because of the high cut-off frequency we have approximated erf$(\sqrt{2\beta} f_c)\approx 1$. Equation (47) is to lowest order proportional to the cross-spectrum of the single spike train and the signal as discussed above. In particular, we find that the filter introduces a low-pass behavior in the cross-spectrum.

Because of its importance for the discussion of the coherence, we present several expressions for the power spectrum in special cases. First, for the qualitative behavior of the coherence for weak stimuli, it suffices to consider the unperturbed power spectrum *S*_SO,SO_ of the SO (no stimulus) which is given by the *n*-fold convolution of the unperturbed power spectrum *S*_*y*0,*y*0_ (obtained from Eq. (45) by setting *D*_*s*_ = 0). Simpler than to carry out the *n* convolution integrals explicitly is to Fourier-transform the *n*-th power of the correlation function *k*_*y*0,*y*0_(*τ*) (i.e. the Fourier transform of *S*_*y*0,*y*0_, which is a constant plus a Gaussian):

$$ \begin{array}{rll} S_{{\rm SO},{\rm SO}} &=& \alpha^2 \int_{-\infty}^\infty d\tau \, e^{2\pi i f \tau} k_{y0,y0}^n(\tau) \\ &=& \alpha^2 r_0^{2n} \int_{-\infty}^\infty d\tau \, e^{2\pi i f \tau} \left[1+\frac{e^{-\frac{\pi^2\tau^2}{2\beta}}}{r_0\sqrt{2 \beta/\pi}}\right]^n \\ &=& \alpha^2 r_0^{2n} \int_{-\infty}^\infty d\tau \, e^{2\pi i f \tau} \sum\limits_{k=0}^n {n \choose k} \frac{e^{-\frac{k\pi^2\tau^2}{2\beta}}}{(2 r_0^2 \beta/\pi)^{(k/2)}} \end{array} $$

which results in the following expression for the power spectrum:

48 $$ S_{{\rm SO},{\rm SO}} = \alpha^2 r_0^{2n-1}\sum\limits_{k=1}^{n} {n \choose k} \frac{1}{\sqrt{k}} \left(\frac{\pi}{2r_0^2 \beta} \right)^{\frac{k-1}{2}}\!\!\!\!\!\exp\left[\frac{-2\beta f^2}{k}\right], $$

where we omitted the DC peak. Now, for a sufficiently small width of the Gaussian with respect to the mean interspike interval, i.e. if

49 $$ r_0 \sigma<\frac{1}{2\sqrt{\pi}}\approx 0.28 $$

(which is met with our standard parameter choice *r*_0_ *σ* = 1/10) and small number of neurons (*n* < 5), one can show that all terms in the sum make significant contributions. (Note that the above condition is identical with Eq. (27) granting a monotonic decrease in the synchronous output rate with growing *n*.) In particular, the exponential

$$ \exp\left[\frac{-2\beta f^2}{n}\right] $$

has the slowest decay in *f* and thus determines the effective cut-off frequency of the power spectrum. Because of this term the power spectrum drops slower than the square of the cross spectrum which leads to an overall low-pass behavior of the coherence.

Using only the leading order terms for both the cross- and power spectrum, we obtain the following expression for the coherence

50 $$ C_{{\rm SO},s}(f) \simeq \frac{n^2 S_{s,s}}{\sum_{k=1}^{n} {n \choose k} \frac{1}{\sqrt{k}r_0^k}\left(\frac{\pi}{2\beta} \right)^{(k-1)/2} e^{2 \beta f^2(\frac{k-1}{k})}}. $$

For comparison to numerical simulations, we also give the expressions for the power spectrum including the first-order corrections due to the stimulus (assuming again high cut-off frequency) for the cases *n* = 2

51 $$ \begin{array}{rll} &&{\kern-6pt} S_{{\rm SO},{\rm SO}}(f) = \frac{r_0^2\alpha^2}{2} \bigg[4(6D_s r_0^2+r_0)e^{-2\beta f^2}+ \sqrt{\frac{\pi}{\beta}} \times\\ &&{\kern-6pt} \left(1+2 r_0 D_s\big[1+\text{erf}(2\sqrt{\beta}(f_c -f/2)) \big]\right)e^{-\beta f^2} \bigg] \end{array} $$

and *n* = 3

52 $$ \begin{array}{rll} &&{\kern-6pt}S_{{\rm SO},{\rm SO}}(f) = \alpha^2 r_0^3 \bigg[ 3r_0(1+10 r_0^2 D_s)e^{-2\beta f^2} + \\ &&{\kern-6pt}+\frac{3r_0}{2}\sqrt{\frac{\pi}{\beta}}\left(1 + 6D_s r_0 \big[1 + \text{erf}(2\sqrt{\beta}(f_c -f/2)) \big] \right)e^{-\beta f^2} \\ &&{\kern-6pt}\frac{\pi/2}{\sqrt{3}\beta}\left(1+3D_s r_0 \big[ 1+ \text{erf}(\sqrt{3\beta}(f_c -f/3))\big] \right)e^{-\frac{2}{3}\beta f^2} \bigg] \\ \end{array} $$

**Fig. 5:**
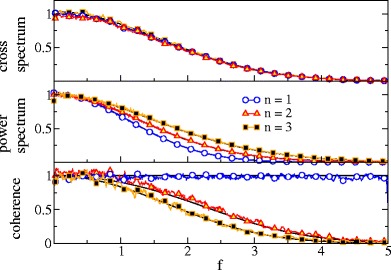
Spectral measures of the SO of Poisson neurons for different population size as indicated. All measures have been rescaled to their global maximum. Theoretical results obtained from same equations as in Fig. 4 except for the expression for the power spectrum for *n* = 3 for which we used here Eq. (52). Parameters: *r*_0_ = 1 and *D*_*s*_ = 0.01, *f*_*c*_ = 5

The spectral measures for single spike trains, summed trains and SO are shown in Fig. 4; here and in Fig. 5 we have used Eqs. (51), (52), (47), and (16) to calculate cross and power spectra, and the coherence function, respectively. As can be seen in Fig. 4, the synchronous output acts as a low-pass filter on information about the stimulus: the coherence of the SO drops with frequency and the cut-off for this drop is roughly set by

53 $$ f\approx \frac{1}{\sqrt{2\beta}}\frac{n}{n-1}= \frac{1}{2\pi\sigma}\frac{n}{n-1}.$$

Although the dependence of the cut-off on *n* is modest, our analytical results confirm the shift to lower values for larger *n* (cf. Fig. 5).

The drop in coherence at higher frequencies is clearly due to the change in the shape of the power spectrum, more specifically, due to the sum over the exponentials with decreasing decay rate. To illustrate this shape change, we consider the SO spectrum *S*_2_(*f*) for *n* = 2 and write the single spike train spectrum as *S*_1_(*f*) = *g*(*f*) + *δ*(*f*), consisting of a symmetric finite-width peak *g*(*f*) around zero (essentially the filter function) and a delta peak (the DC peak) *δ*(*f*) (setting the rate for simplicity to one). Then, to lowest order the SO power spectrum reads

54 $$ S_2 = S_1 * S_1 = g * g(f) + 2 g(f) +\delta(f). $$

A symmetric finite-width peak around zero, if convolved with itself, becomes broader, so the first term turns in our example of a Gaussian filter into a Gaussian with twice the variance. There is, however, also a term proportional to the original peak and there is also the DC peak left. Another convolution with *g*(*f*) + *δ*(*f*) will yield for *S*_3_(*f*) all combinations of Gaussians with unit, double, and triple variance and also a delta function.

Our interpretation of the low-pass filtering of information is as follows. The operation by which we define the SO (Gaussian filter and multiplication) comes along with a loss of temporal precision and thus with a diminished information transfer at higher frequencies. Decreasing *σ*, the width of the filter, would push the cut-off frequency to higher values, although it would also result in a substantial overall reduction of coherence because of the decrease in the SO firing rate.

We also would like to point out that the Poisson model is unable to reproduce the experimental finding of a SO coherence maximum at finite frequency - the maximum of the coherence of the SO of a Poisson population is always at zero frequency. We confirmed this finding also using another filter, the box filter and the method by Middleton et al. (2009) involving the reference spike train.

### Population of leaky integrate-and-fire neurons

As a second and more realistic neuron model we use the leaky integrate-and-fire (LIF) neuron driven by a white noise current and by a current stimulus. The subthreshold voltage of this stochastic LIF model, i.e. of the *k*th neuron of our population obeys the well-known dynamics

55 $$ \dot{v}_k=-v_k+\mu+s(t) + \sqrt{2D_i}\xi_k(t) , $$

where *μ* is the constant base current, *ξ*_*k*_(*t*) is a Gaussian white noise with zero mean and correlation function $\langle{\xi_k(t)\xi_{k'}(t')}\rangle=\delta_{k,k'} \delta(t-t')$, and *D*_*i*_ is the intrinsic noise intensity; time is measured in units of the effective membrane time constant. Note that the intrinsic white-noise sources *ξ*_*k*_(*t*) are completely uncorrelated among each other, whereas the stimulus is common to all neurons. Whenever the voltage crosses the threshold *v*_*t*_, a spike is generated and the voltage is reset to the value *v*_*r*_. In this paper we work with a nondimensional rescaled voltage, for which we can choose *v*_*r*_ = 0 and *v*_*t*_ = 1 without loss of generality (Vilela and Lindner 2009a).

Because both intrinsic fluctuations and stimulus are modeled as white Gaussian noise, we will also refer in the following to the total noise intensity

56 $$ D=D_i+D_s . $$

The dynamics of a single LIF neuron under white noise stimulation and its response to weak signals has been reviewed in a number of papers (Fourcaud and Brunel 2002; Burkitt 2006a, b), in particular, its spectral coherence function has been inspected and compared to those of other integrate-and-fire neurons (Vilela and Lindner 2009b). Here we need some formulas for the single white-noise driven LIF and will thus state them briefly in the following.

The firing rate *r*_0_ in the absence of a signal is (Ricciardi 1977)

57 $$ r_0 = \left[\sqrt{\pi} \int_{(\mu - v_t)/\sqrt{2D}}^{(\mu - v_r)/\sqrt{2D}} dz \, e^{z^2} \mbox{erfc}(z)\right]^{-1}. $$

The background spectrum, which is the power spectrum of a single spike train in the absence of a signal, can be expressed by (Lindner et al. 2002)

58 $$ \begin{array}{rll} S_{x_0,x_0}(f) &=& r_0 \frac{| {\cal D}_{2\pi i f} (\frac{\mu-v_t}{\sqrt{D}})| ^2 - e^{2 \Delta}| {\cal D}_{2\pi i f} (\frac{\mu-v_r}{\sqrt{D}})| ^2}{| {\cal D}_{2\pi i f} (\frac{\mu-v_t}{\sqrt{D}})-e^{\Delta} {\cal D}_{2\pi i f} (\frac{\mu-v_r}{\sqrt{D}})|^2} \\ && +\, r_0^2\delta(f), \end{array} $$

where

59 $$ \Delta = \frac{v_r^2-v_t^2+2\mu (v_t-v_r)}{4 D}, $$

and ${\cal D}_a(z)$ is the parabolic cylinder function (Abramowitz and Stegun 1970). The susceptibility *χ*(*f*) of the white-noise driven LIF neuron is (Lindner and Schimansky-Geier 2001) (for alternative expressions in terms of hypergeometric functions, see Fourcaud and Brunel (2002) and references therein)

60 $$ \chi(f) = \frac{r_0 2 \pi i f/\sqrt{D}}{2\pi if-1} \frac{{\cal D}_{2\pi if-1}(\frac{\mu-v_t}{\sqrt{D}})-e^{\Delta}{\cal D}_{2\pi if-1}(\frac{\mu-v_r}{\sqrt{D}})}{{\cal D}_{2\pi if}(\frac{\mu-v_t}{\sqrt{D}})-e^{\Delta} {\cal D}_{2\pi if}(\frac{\mu-v_r}{\sqrt{D}})} . $$

For the theoretical results on spectral measures, we use in the following numerical evaluations of the integrals involving convolutions of power spectra and susceptibility, i.e. there are no further simplifications possible because the appearing integrals are intractable analytically.

**Fig. 6:**
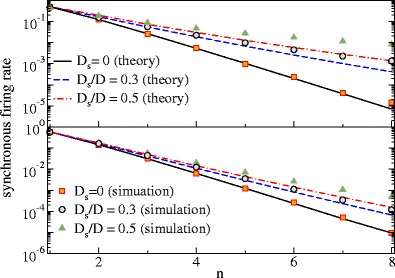
The mean synchronous output (‘synchronous firing rate’) of LIF neurons as a function of the population size *n* for different signal intensities as indicated. Parameters: *μ* = 0.8, *D* = 0.2 (*upper panel*); *μ* = 1.2, *D* = 0.01 (*lower panel*)

First, we consider the mean output. Similar to the Poissonian case, the synchronous firing rate drops with the population size and increases with the intensity of the common stimulus (Fig. 6) as predicted by theory. The curves for different stimulus variance give us also an idea about the range of validity of our linear response assumption—deviations (theory underestimating the simulation results) are seen if the signal makes up about 30% of the stochastic driving and are even more severe for a 50% contribution, for both sub- and suprathreshold base current (upper and lower panel in Fig. 6).

The firing statistics of the LIF neuron depends strongly on the parameter regime (Fourcaud and Brunel 2002; Burkitt 2006a; Lindner et al. 2002; Ostojic 2011). For small subthreshold *μ* (*μ* < *v*_*T*_) and small noise, the spontaneous spiking approaches a Poisson process, i.e. the coefficient of variation of the interspike intervals, *C*_*v*_, is close to one and the interspike interval density is nearly exponential. For strong *μ* (*μ* > *v*_*T*_) and low noise, in contrast, the firing is rather regular with a low *C*_*v*_ and a histogram sharply peaked around the mean ISI. The regime of very strong noise is yet another case, in which the *C*_*v*_ can become much larger than one and the voltage can perform long excursions towards negative values before reaching the threshold (this case might be the biological least relevant on physiological grounds). We do not expect that the coding of a common stimulus by synchronous spikes shares the same properties in all these regimes and inspect them therefore separately.

**Fig. 7:**
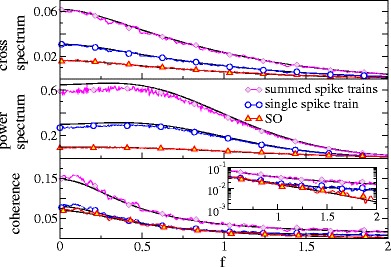
Spectral measures of the single spike train, the summed spike train, and the SO for LIF neurons in the subthreshold (noise-induced) firing regime. Parameters: *n* = 2, *μ* = 0.8, *D* = 0.2 and *D*_*s*_ = 0.02

**Fig. 8:**
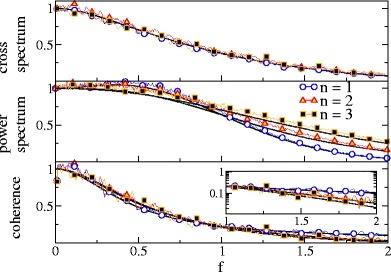
Spectral measures of the SO for LIF neurons in the subthreshold (noise-induced) firing regime for different numbers of neurons as indicated. All curves are rescaled to their respective maximum. Parameters: *μ* = 0.8, *D* = 0.2 and *D*_*s*_ = 0.02

For subthreshold base current *μ* = 0.8 and moderate noise *D* = 0.2, where spiking is rather irregular (*C*_*v*_ = 0.73), we observe a low-pass coherence function for single and summed spike train and SO (cf. Fig. 7). The LIF model on its own, however, shows already some low-pass behavior with respect to coherence (Vilela and Lindner 2009b). We observe an additional low-pass effect on the synchronous output, which becomes apparent when comparing the SO coherence to that of the single spike train as is done in the inset of Fig. 7 (lower panel). This is similar to the model of Poisson neurons in the previous section. For the latter we also observed that by increasing the number of spike trains, the effective cut-off frequency of the coherence attains lower values. The same effect is found for increasing the number of LIF spike trains in the SO (Fig. 8): for *n* = 3 the effective cut-off occurs at a lower frequency than it does for *n* = 2 as becomes best visible on a logarithmic scale (inset of Fig. 8, lower panel).

**Fig. 9:**
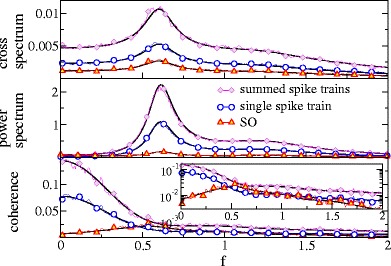
Spectral measures of the single and summed spike trains, and the SO for LIF neurons in the suprathreshold (deterministic) firing regime. Parameters: *n* = 2 *μ* = 1.2, *D* = 0.01 and *D*_*s*_ = 0.001

As shown in Fig. 9, a different behavior is observed for the LIF in the deterministic firing regime with a base current *μ* = 1.2 and a weak noise *D* = 0.01 resulting in a rather regular firing (*C*_*v*_ = 0.24). Here, power (Fig. 9, upper panel) and cross-spectra (Fig. 9, middle panel) are all peaked around a frequency corresponding to the firing rate (see e.g. Knight (1972a; Fourcaud and Brunel (2002) for the cross-spectral peak and Lindner et al. (2002) for the peak in the power spectrum).

For the single and the summed spike train, both peaks cancel in the coherence, which decays monotonically with frequency. In contrast, for the SO the peaks in the squared cross spectrum and power spectrum are sufficiently different to still result in a peak in the coherence function around the firing rate. This maximum of the SO coherence vs frequency is attained at finite frequency and is thus qualitatively similar to the one observed in experiment (see Fig. 3(c) by Middleton et al. (2009)). The maximum is more pronounced for a larger population of spike trains.

**Fig. 10:**
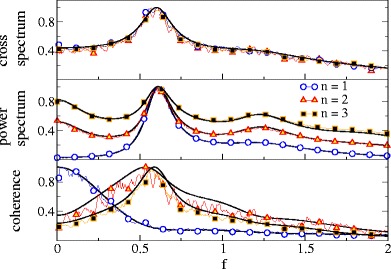
Spectral measures of the SO for LIF neurons in the suprathreshold (deterministic) firing regime for different numbers of neurons as indicated. All curves are rescaled to their respective maximum. Note that while cross-spectra are very similar, the width of the spectral peak in the power spectra depends on *n* and because of the increasing width, the coherence for *n* > 1 shows a maximum vs frequency. Parameters: *μ* = 1.2, *D* = 0.01 and *D*_*s*_ = 0.001

In Fig. 10 we inspect whether the qualitative change in the coherence function is due to the cross- or due to the power spectra. The frequency dependence of the cross spectrum does not change significantly (apart from a change in magnitude that has been compensated by rescaling each curve by its maximum) upon increasing the population size, see Fig. 10, upper panel. The power spectrum, in marked contrast, changes drastically: the more spike trains are used to define the synchronous output, the broader becomes the peak around the firing rate, further, the larger *n*, the stronger becomes the peak at zero frequency. This change in the power spectrum is evidently responsible for the peak in the coherence function.

To lowest order, the power spectrum of the SO is given by an *n*-fold convolution of the spontaneous power spectrum with itself. To see what happens to a peaked power spectrum upon convolution with itself, we consider a generic spectrum of the form

61 $$ S(f)=\hat{t}_- g(f)+ r_0^2 \delta(f)+\hat{t}_+ g(f) $$

where *g*(*f*) is a function with a finite-width peak at *f* = 0, $r_0^2\delta(f)$ is the DC peak of the spectrum, and $\hat{t}_\pm$ are shift operators that shift the function by the firing rate *r*_0_. Convolving this spectrum with itself and using the translation invariance of the convolution, we obtain

62 $$ \begin{array}{rll} &&S_2(f)=S * S (f) \\ &&= (\hat{t}_- g+r_0^2 \delta(f)+\hat{t}_+ g)* (\hat{t}_- g+r_0^2 \delta(f)+\hat{t}_+ g) \\ &&= \hat{t}_-^2 (g* g)+\hat{t}_+^2 (g* g)+2 r_0^2[\hat{t}_- g+\hat{t}_+ g] \\ && \;\;\;\;\;\; +\, r_0^4 \delta(f)+ 2 (g* g), \end{array} $$

where we have used that the shift operator commutes with the convolution and that $ \hat{t}_-\hat{t}_+= \hat{t}_+\hat{t}_-=1$. Restricting ourself to positive frequencies, the spectrum *S*_2_ shows finite-width peaks at (1) the firing rate; (2) twice the firing rate (a higher harmonics); (3) at zero frequency. The latter two peaks are proportional to g* *g* and thus have an increased width. These peaks contribute to increase the effective width of the summed peak around *r*_0_. Hence, it is the nonlinear operation of multiplication leading to higher harmonics and peaks at zero frequency that shape the power spectrum and increase effectively the width of the peak around the firing rate.

**Fig. 11:**
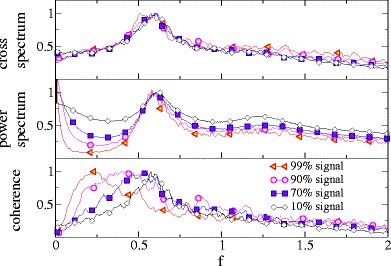
Spectral measures of the SO for LIF neurons in the suprathreshold (deterministic) firing regime for different strength of the stimulus. Parameters: *n* = 3, *μ* = 1.2 *D* = 0.01

In Fig. 11, we show simulation results for the case of very strong stimulation. In the deterministic firing regime (e.g. *μ* = 1.2) the spectral measures are still peaked around the firing rate and a peak in the coherence is obtained. However, the peak frequency moves to smaller values and the coherence curve of the SO approaches the coherence curve of the summed spike train from below.

Because in the paper by Middleton et al. (2009) the peak of the coherence did not appear around the firing rate but at lower values, we conclude that the stimulus amplitude in the experiment were chosen in the nonlinear response regime of the sensory cell.

**Fig. 12:**
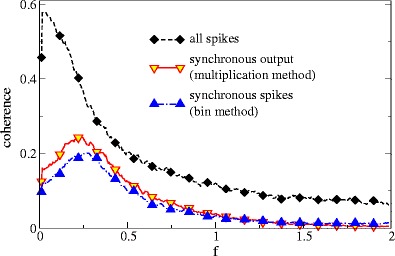
Spectral measures of the SO and the summed spike train for two LIF neurons (*n* = 2) compared for different definitions of the SO: coherence for the product of Gaussian convolutions (multiplication method) with *σ* = 0.26 and coherence for the SO obtained from coincidences of the other spike train with the reference spike train (bin method) in a time bin of width 0.89. Other parameters: *μ* = 0.95, *D* = 0.014 and *D*_*s*_ = 0.0084

Last but not least, we compare in Fig. 12 the peaked SO coherence to the coherence of the synchronous output as it was defined by Middleton et al. (2009). The criterium for the width of the time window was here that a similar mean value of the synchronous output was achieved; the respective time window was Δ*t* = 0.89 in our nondimensional time units, which compares well to the value used for the experimental data by Middleton et al. (2009) if we assume a membrane time constant of 1 ms (Chacron 2006).

Although peak values of the coherence differ somewhat for the two distinct definitions of the SO, the overall shape and, in particular, the location of the peak coincides. This suggests that our results (derived for the product of Gaussian convolutions) are robust and hold true also for slightly different definitions of synchronous activity for populations of neurons.

We also note that although the coherence of the SO never becomes larger than that for all spikes, it can become larger than the coherence of a single spike train—see e.g. how the red line exceeds the blue one in a small intermediate frequency band in the lower panel of Fig. 9. This is at the first glance surprising because we essentially obtain the synchronous output by deleting spikes from a reference spike train which seems to necessitate a reduction in information. However, this reduction is applied according to the firing of the remaining *n* − 1 neurons, hence, in part according to what the stimulus does. It is not hard to see that by deleting spikes from a spike train in a signal-dependent way, we can make (even a previously stimulus-unrelated) spike train carry information about the stimulus. That is why it is meaningful to compare the coherence of synchronous activity to both the coherence of all spikes generated in the population as well as to the coherence of the single neuron.

## Summary and conclusions

In this paper, we have developed a framework for the analytical study of coding properties of synchrony in neural populations. We derived general formulas for stimulus transfer and noise spectrum of the synchronous output of a homogeneous population of neurons which are driven by a common stimulus. By choosing a rather strict multiplicative synchrony criterion we were able to use a linear response ansatz to calculate spectral spike train statistics for single, summed, and synchronous outputs. As we have shown by comparison to numerical simulations of populations of Poisson and LIF neurons, this theory yields reliable results as long as the stimulation is weak.

We were interested under which circumstances the synchronous output selectively encodes certain features of a stimulus. For this to occur, a first condition to be met is certainly that the population has a noteworthy synchronous output at all. By analyzing the mean synchronous output (Eq. 25 and Figs. 3 and 6), we found that for small numbers of neurons and moderate common stimuli the mean is an appreciable fraction of the firing rate (mean of the spike train) of a single neuron.

For the case of a sufficiently strong synchronous output, we then asked whether its coherence with the signal can show a maximum vs frequency as suggested by the experiments of Middleton et al. (2009). We found that for Poisson neurons and for LIF neurons in the subthreshold regime with weak noise (irregular firing with a *C*_*v*_ not too far from one), the coherence of the synchronous output decreases with frequency. This effect is caused by a shaping of the power spectrum of the synchronous output. While the transfer function of the synchronous output does not change qualitatively by the multiplication operation (it is proportional to that of the single cell), the power spectrum becomes broader. Hence, for neurons with rather irregular firing pattern (*C*_*v*_ ≈ 0.7 − 1.3), the coherence of the synchronous output did not show a peak at finite frequency and thus synchronous spikes carry qualitatively the same kind of information as all spikes.

However, for LIF neurons with a pronounced peak at finite frequency in both power- and cross-spectra, we found a different effect. While the transfer function is again unchanged, the nonlinear operation of multiplication of spike trains, that we use to compute the synchronous output, gives rise to new peaks in the power spectrum. These new peaks broaden the peak around the firing rate and via the denominator of Eq. (16) suppress the coherence at low and high frequencies but not around the firing rate.

What are the conditions for a peak of the coherence of the synchronous output at finite frequency? Apparently, the power spectrum of the single cell has to have a finite-width peak at finite frequency.This is the case for an LIF neuron with a suprathreshold base current (in our setting *μ* > 1) and with small to moderate noise intensity *or* for a subthreshold base current (*μ* < 1) and a properly tuned noise intensity (so-called coherence resonance effect as demonstrated in the LIF neuron e.g. by Pakdaman et al. (2001) and Lindner et al. (2002)). We have verified that indeed a maximum in the coherence can be observed for a subthreshold base current (e.g. *μ* = 0.9) and a suitable noise intensity causing a peak in the power spectrum. We also note that factors like adaptation (Benda and Herz 2003), threshold fatigue (Chacron et al. 2007), or bursting (Bair et al. 1994) shape the response function and the power spectrum (leading to more pronounced spectral peaks, see e.g. (Lindner and Longtin 2003)) and may thus influence the information filtering seen in the synchronous activity.

Although a peaked coherence is observed only in a regime of rather regular firing, the firing must not be “too regular”, i.e. the effect hinges upon the presence of a sufficient amount of intrinsic fluctuations. Certainly, in the absence of internal noise, all neurons fire phase-locked with respect to the stimulus (Knight 1972a) and the synchronized output converges to the spike train of a single neuron, which possesses a low-pass coherence. Our results indicate that for weak stimulus and sufficient intrinsic noise, new peaks in the power spectrum of the synchronous activity have to be *broad enough* to make an impact on the peak around the firing rate. Because these peaks arise from the convolution of the former peak, this requires that also the original peak is not “too thin”. Coming from this weak-stimulus-and-moderate-intrinsic-noise scenario and increasing either the stimulus amplitude or decreasing the intrinsic noise (both was done at the same time in Fig. 11), the maximum of the coherence moves towards zero, and the coherence of the synchronous output becomes more similar to both the coherence of the single spike train and the coherence of all spikes. In other words, the intrinsic noise as well as the stimulus amplitude control the best frequency of the coherence of the synchronous output. The maximal best frequency possible seems to be the firing rate. In order to generate an information filter with this frequency, the intrinsic noise should not be too weak (keeping a finite width of the spectral peak and linearizing the transfer), but also not too strong (keeping a peak at finite frequency at all). For a given stimulus amplitude there should be an optimal noise strength in order to establish an information bandpass-filter that employs the synchronous output.

There is another parameter of interest in our model— the width *σ* of the Gaussian filter. In real neurons, the time-scale of the filter could correspond to the width of the postsynaptic potential. According to our results, coding by synchrony is meaningful only if this postsynaptic time scale is much smaller than the mean ISI of each presynaptic cell. In addition, we expect a peaked coherence only if these two time-scales are sufficiently separated because a too broad filter destroys the peaked structure of cross- and power spectra on which the information filtering is based.

Note also that we defined the synchronous output as the multiplication of the spike trains convolved with a Gaussian filter. This allowed us to derive expressions for the coherence of the synchronous output but at the same time is a rather strict notion of synchrony. Every neuron in the population needs to fire a spike within the width of the filter to result in a non-zero synchronous output. This notion of synchrony compares well to the binning method for two spike trains (Fig. 12) as used for analyzing the data by Middleton et al. (2009). However, for populations larger than a few neurons this definition of synchrony results in vanishing output rates. The more realistic situation is a much larger population of neurons that converges onto a target neuron and that the synchronous activity of only a certain fraction of the neurons is sufficient to trigger an action potential in the target neuron. This would result in higher synchronous outputs. Simulations show that in such a case the response of the target neuron has a peak in the coherence as well (Middleton et al. 2009), demonstrating that our results translate to larger populations of neurons with a softer synchrony criterion.

Our theory generally demonstrates that by solely reading out synchronous spikes from a population of neurons receiving a common stimulus a target neuron can selectively extract specific features of the stimulus, namely those frequency components that are close to the firing rate of the input population. This is in marked contrast to the much more broad-band coherence between all spikes of such a population and the stimulus. The non-linear operation of reading out synchronous spikes thus processes the information about the stimulus that is contained in the spiking activity of a population of neurons. In addition, this offers the possibility of a parallel readout of different levels of synchrony by different target neurons in order to extract different aspects of the encoded common stimulus.

In the electrosensory system of weakly electric fish electric stimuli are encoded by a population of electroreceptors. The receptor afferents project to pyramidal cells in three parallel maps. There is experimental evidence that in the map where the pyramidal cells have a peaked coherence function (Krahe et al. 2008) the pyramidal cells indeed read out synchronous spikes by coincidence detection from about one thousand afferents (Maler 2009). In another map, where only about ten afferents converge onto a target cell, the pyramidal cells show a broad-band coherence and presumably decode all input spikes (Middleton et al. 2009). Since this effect is a quite general property of populations of spiking neurons we expect it to be employed in many other sensory systems as well. In higher processing stages of the brain, however, where neurons have low firing rates and operate in their fluctuation driven regime reading out different levels of synchrony does not yield different aspects about the signal anymore.

Our analytical approach can be also extended to other filter functions, to heterogeneous populations of neurons, and to populations, in which only a fraction of cells receives the common stimulus. A more advanced generalization of the theory concerns populations of coupled neurons instead of neurons that are only synchronized by a common drive. In the coupled case, the shaping of the information transfer in the synchronous activity may be even more severe. These are exciting problems for future studies on information transfer in neural populations.
